# Facilitators’ experiences with virtual simulation and their impact on learning

**DOI:** 10.1186/s41077-024-00323-1

**Published:** 2024-12-31

**Authors:** Margaret Verkuyl, Efrem Violato, Theresa Southam, Mélanie Lavoie-Tremblay, Sandra Goldsworthy, Diane MacEachern, Lynda Atack

**Affiliations:** 1 School of Community and Health Studies, Centennial College, P.O. Box 631 Station A, Toronto, ON Canada; 2 Northern Alberta Institute of Technology, 11762-106 Street, Edmonton, AB T5G 2R1 Canada; 3 Teaching and Learning Centre, Selkirk College, 301 Frank Beinder Way, Castlegar, BC V1N 4L3 Canada; 4 Faculty of Nursing, Centre de Recherche de l’Institut Universitaire en Santé Mentale de Montréal, University of Montreal, 2375, chemin de la Côte Sainte-Catherine, Bureau 2089, Montréal, QC H3T 1A8 Canada; 5 School of Nursing and Midwifery, Mount Royal University, 4925 Mount Royal Gate SW, Calgary, AB T3E 6K6 Canada

## Abstract

**Background:**

Increasingly, virtual simulations are being integrated into higher education. A successful experience goes far beyond simply offering learners access to a virtual simulation; it requires a facilitator who understands the learners’ needs and course objectives, choses the right virtual simulation for the learner, creates a welcoming space that promotes learning, and evaluates the experience.

**Methods:**

Facilitators from three different healthcare programs and six educational institutions and students from two different healthcare programs were included in this exploratory qualitative research study. Interviews and focus groups and thematic analysis were conducted to understand the role of the facilitator when using virtual simulations and their impact on student learning.

**Results:**

The facilitator themes were supported by the student focus groups. The first theme, the facilitator experience, included sub-themes of simulation pedagogy and debriefing with a practice partner. The second theme was virtual simulation: impact on learning and included sub-themes on student outcomes, technology and design, and repetitive play.

**Conclusion:**

Effective facilitation skills are integral to quality virtual simulation experiences. Trained facilitators help students achieve virtual simulation learning outcomes and prepare for clinical practice.

## Highlights

• Trained facilitators are crucial to an effective virtual simulation experience.

• Applying simulation pedagogy positively affects student outcomes.

• Practice partners enhance the debriefing experience.

Virtual simulation (VS), while a relatively new modality in the field of education, rapidly gained momentum during the Covid-19 pandemic. The *Healthcare Simulation Dictionary* describes VS as the recreation of reality portrayed on computer screens, involving real people operating simulated systems and playing key roles in performing skills, engaging in decision-making, or communicating [18]. While the pandemic was a major factor in VS uptake, cost, and scheduling challenges of in-person simulation [3], the challenge of finding sufficient quality clinical placements and a growing interest in providing students with workplace-integrated learning opportunities that develop their employability skills is also driving VS adoption [13]. A large body of literature suggests that VS is an effective, experiential learning tool [3], which can develop knowledge, skills, and critical thinking [10], and realistically represents work-integrated learning experiences and prepares students for the workplace [14, 31]. Diaz et al. [8] found no significant difference between high fidelity and VS on knowledge outcomes and concluded that VS can be safely used to replace some high-fidelity, in-person simulation and clinical hours.

While much is known about students’ experiences and outcomes with VS, little is known about what is required to effectively conduct the complex activity of facilitating simulation in the virtual environment [16], nor the experiences of facilitators. For this paper, we followed the *Healthcare Simulation Dictionary* definition of facilitator (Simulation Facilitator) as “An individual who is involved in the implementation and/or delivery of simulation activities” (p.18, [18]). This means that facilitation is more than simply offering learners access to a VS; it requires facilitators trained in the implementation and delivery of simulations. A quality VS experiences requires facilitators who understand the learners’ needs and course objectives, chooses the right VS for the learner, creates a welcoming space that promotes learning, and evaluates the experience. Several authors have noted the importance of sound facilitation practices in VS related to best practices [5, 24, 26, 27]. Violato et al. [30] and Park et al. [22] found that simulation best practices related to facilitation are often not described in the VS literature with Park et al. noting that facilitators are often clinical experts who may have been trained in simulation but not in VS facilitation. Badowski and Wells-Beede [1], in a study of nurse educators teaching with VS, noted that less than half of the participants had formal training in both prebriefing and debriefing, and 10% had no formal training in debriefing. These authors questioned how learning objectives could be met when VS was conducted by untrained debriefing facilitators. Holmes et al. [12] in a review of the literature related to facilitation of interprofessional simulation noted that in addition to facilitation skills, the clinical credibility of the facilitator played an important role in student learning. In our extensive experience facilitating VS, we have noted that facilitation skills used with in-person simulation do not completely transfer to the virtual world. In the in-person simulation experience, all stages are done face to face, however, with VS, facilitators make choices about whether to enact the VS as a group or whether to ask students to complete the experience on their own. Depending on the enactment, facilitators also need to make good decisions regarding the debriefing modality. Students can certainly learn from self-debriefed VS; however, one study concluded that self-debriefing resulted in lower debriefing satisfaction scores [28]. In other another study of healthcare students, combined debriefing (self-debrief and facilitator-led group debrief) improved communication skills and team effectiveness when compared to self-debriefing alone [25]*.* A study by Morton et al. [21] on learner and facilitators’ perceptions of engagement in virtual debriefs highlighted the need for strategies to help facilitators debrief virtually so they can engage their learners. In another study, Cheng et al. [5] provided practical tips on enhancing social, teaching, and cognitive presence in a virtual debrief. Violato et al.’s [30] scoping review highlighted the importance of not assuming that in-person best practices are obviously transposed to the VS environment, as with any new method, evidence needs to be gathered, particularly regarding VS facilitation. While research in VS facilitation is advancing, an important gap persists in our understanding of the best ways to facilitate the different modalities used in VS and what skills, professional development, experience, and supports facilitators need.

Colleges and Institutes Canada (CICan), in collaboration with Simulation Canada, and with colleges, institutes, and universities across the country, has offered students innovative work-integrated learning opportunities using VS through the Virtu-WIL (work-integrated learning) initiative. The Virtu-WIL program addresses clinical placement challenges by creating new and innovative virtual ways for healthcare students in nursing, medical laboratory sciences, and paramedicine to acquire competencies and make valuable connections with employers. The VSs, created by educators across Canada, are simulated clinical experiences based on defined pedagogical objectives. They are freely available to students and faculty globally (see https://simulationcanada.ca/virtu-wil/).

In 2023, at the time of the study, Canadian educators and students across the country had access to 137 VSs in the areas of nursing, medical lab technology, and paramedicine, through the Virtu-WIL program. Six different platforms delivered the 137 different VS that were available to facilitators. Some platforms were easy to navigate, such as Affinity, while others such as PCS Spark were more complex due to the natural language software and room navigation. For this program, facilitators chose three to five VS from the repository that their students were required to play, meaning they often facilitated VS on different technology platforms. They also organized and held the prebrief and debrief sessions. Approximately, 2850 students were enrolled in the program in the past 12 months. The facilitatators were trained faculty in the specific program areas. Some facilitators had previous simulation training and experience, while others had no prior simulation experience. All facilitators were provided with an opportunity to learn about VS and how to facilitate VS experiences through five online self-study modules, which each took approximately two hours to complete. They were also given access to peer-reviewed facilitation guides for each VS. To strengthen the connection between the VS experience and the workplace, clinical staff from placement sites were invited to provide content expertise in the debriefing sessions.

The purpose of this study was to answer two research questions:

1. How well prepared were facilitators in the Virtu-WIL project, i.e., what were the facilitators’ perceptions of their training needs and what recommendations did they have for training?
2. From a student and a facilitator perspective, what was the impact of the VS on student learning?

## Material and methods

### Ethics

The study was reviewed and approved by the participating authors’ Research Ethics Boards institutions as required (Centennial College no. 2022/23–10 & George Brown College no. 6004781 & University of Nipissing no. 103213). Participation was voluntary, and participants could withdraw their data at any time unless it was a group interview. Participants gave informed consent. Once tapes were transcribed, any identifying information was removed, and the tapes were erased.

### Design

We used an exploratory qualitative research process described by Hodges et al. [11]. Hodges et al. conducted a national scan to explore issues and challenges related to future medical education. Because we had similar goals as Hodges et al., exploring innovation in healthcare education and factors facilitating and hindering that innovation, their approach was a good model for our study. Our review of the literature suggested there was still much to be learned about the complex act of facilitating [7] and the factors that influence facilitation, therefore, Hodges et al.’s inductive, exploratory approach aligned with our study goals. We modified the Hodges et al. process in that we conducted a more focused study with group and individual interviews rather than conducting a wider environmental scan. We opted for focus groups, where possible, as they can stimulate discussion, elicit a range of views, and may offer a sense of security to participants [20]. For practical reasons, such as scheduling constraints, the facilitator interviews, and one student interview, were conducted individually.

Our goal was to hear diverse opinions on our research topic. As with Hodges et al., we started by using stratified sampling, a subset of purposive sampling, to ensure that we interviewed (a) participants from different groups (facilitators and students), (b) from different educational institutions, and (c) different healthcare programs across Canada. That sampling approach aligned with our research objective which was to capture different views and experiences of VS from the different participant groups [4]. Potential participants were contacted by the project assistant by email with information regarding the study, noting that participation was completely voluntary.

We aimed to conduct three to four focus groups with students and facilitators from three programs: nursing, paramedicine, and medical laboratory technology. Once we had secured program consent to participate from different institutions, we used convenience sampling at those institutions to build our sample. The focus group and individual interviews were held in 2023 with students and facilitators from the participating programs.

### Data collection and analysis

A semi-structured interview guide was used to conduct the interviews (Tables 1 and 2). Semi-structured interviews guide the interview; however, because the interviewer can modify the sequence and wording of the interview questions, this approach is very effective at tapping into the participant’s experience [6].

**Table 1:** Facilitator interview guide

1. What, if any, prior experience has you had with facilitating a virtual debrief?
2. Did you feel well prepared to facilitate the virtual debriefing? Can you think of anything that would have helped to better prepare you to facilitate the virtual debriefings with students?
3. From your observations during the debriefings, do you think that the VSs had an impact on student learning?
4. In your opinion, what was it about the VS design or learning process that facilitated learning?
5. Do you think the VSs influenced your students’ readiness for clinical practice? If no, can you tell me more? If yes, can you give me some examples?
6. Was a clinical partner (i.e., nurse, lab technician) present with you during the debrief? If so, what role did they play and what impact do you think that had on student learning? Do you think it prompted a readiness for practice? Were there other benefits to working with a clinical partner during the VSs? Would you recommend having a clinical partner present at future debriefings?
7. Do you have any recommendations you’d like to share regarding using VSs to enhance student’s clinical practice?

**Table 2:** Student interview guide

1. How many virtual simulations did you play? Do you play any of them more than once, if so, how many times? Were there any barriers to your ability to access/play the virtual simulations?
2. Can you tell me about your experience with the virtual simulations you played as part of the Virtu-WIL program?
3. Were the virtual simulations you played helpful to your learning? If so, what do you think you learned by using virtual simulation? Can you give me an example?
4. I’m going to ask you now to think about your clinical practice, after using the virtual simulations. Did the virtual simulation help to prepare you for clinical practice? If no, can you tell me about that? If yes, can you give me an example of how the VS helped you prepare for clinical practice?
5. Thinking about the virtual simulations, do you have any examples of how they influenced your actual clinical practice? Do you have any other examples of how the simulations influenced your clinical practice?
6. In your opinion, what was it about the VS design or learning process that helped you prepare for clinical?
7. Was someone from practice present during your debrief (i.e., a nurse, lab technician)? If so, what role did they play?
8. Do you have any recommendations you’d like to share regarding using virtual simulations to enhance your clinical practice?

The interviews were conducted using a web-conferencing site and were approximately 30 min in duration for individuals and 60 min for groups. The interviews were recorded and transcribed, and once transcribed, the interviews were erased. The sessions were moderated by researchers experienced with facilitation who were familiar with the VSs. We, the research team, used a thematic content analysis approach as described by Hodges et al. [11]. Two members read through all the transcripts and extracted major codes. The full team then reflected on the codes and used them as a guide as they read through all the transcripts. Next, at a web-conferencing session, the codes were revised, refined, and validated. Any original codes that did not hold up were discarded, while any new major codes were added, and transcripts were re-reviewed. We searched the data for negative/nonpositive examples. Once coding was completed, the team reviewed each transcript again, identifying the themes and sub-themes in the transcripts and any participant statements that effectively illustrated the theme.

We maintained rigor throughout the sampling, data collection, and analysis states. Rigor was maintained by being mindful that we had our own opinions on facilitation; as such, we chose an exploratory research design to seek out others’ experiences. We also kept notes on institutions and programs that had been invited to participate, ensuring we had representation from the different health care programs under study as well as a range of participating institutions. Researcher triangulation was also employed in both data collection and analysis. Researchers who conducted the interviews were experienced with VS, and they reviewed the guide together and discussed what prompts and probes would be used. We developed an interview guide with open questions that did not “lead” the participants. We examined the data as they were collected and started thematic analysis after the first few interviews from each group were conducted. This process helped us to recognize repeating themes and to identify when very little new information was coming in. We then conducted a further three interviews from each group to help achieve data saturation. Regular peer debriefings with the entire team through the analysis process enhanced the rigor of this process. This careful attention to detail enhanced the credibility of the data collection and analysis process.

### Reflexivity

The principal investigator has extensive experience with developing, teaching with, evaluating and facilitating VS. Other members of the research team were also experienced in these different fields related to VS. Our experiences were instrumental in developing the research and the interview questions. We were mindful of our experiences, both positive and negative, when analyzing and reporting the data and used a critical, triangulated approach as described in the “Data collection and analysis” and “Discussion” sections of this paper.

## Results

Ten facilitators participated in the study: three from nursing, three from medical lab, and four from paramedicine from six different educational institutions. There were five student focus groups involving 21 students from five institutions: eight from paramedicine and 13 from nursing. Two themes were identified: the facilitator experience and virtual simulation: impact on learning (Table 3). In this section, reports from the facilitator interviews are indicated by F, and the student focus groups are indicated by FG.

**Table 3:** Themes and sub-themes

**Facilitator experience**
Simulation pedagogy
Debriefing with a practice partner
**Virtual simulation: impact on learning**
Student outcomes
Technology and design
Repetitive play

### The facilitator experience

#### Simulation pedagogy

Two sub-themes were identified under this major theme: Simulation pedagogy and debriefing with a practice partner. Facilitators and students were clear: to be effective, VSs need to align with course learning objectives, meet learner needs, and be skillfully facilitated. One facilitator (F2, nursing) noted, “It just comes down to the skill of the people you've got working with the students facilitating with them.” A skilled facilitator plans and applies simulation pedagogy through all stages of the VS, the preparation, prebriefing, enactment, and debriefing. Facilitators told us that the preparation and prebrief stages are important to prepare students for learning. Facilitators emphasized the particular importance of the debriefing.

The quality of the debriefing is enhanced when facilitators have access to carefully developed, peer-reviewed facilitator guides; however, the success of facilitator guides depends on the experience of the person doing simulations and facilitating debriefs. Facilitators need to view the guides as basic templates and adapt them to their students’ levels and needs. “I think there is a real art to guiding and debriefing in a way where students feel comfortable speaking and expressing their experiences with the simulation (F9, medical lab).” Some facilitators indicated they would have liked more preparation and training. Facilitators also noted that debriefing is best done in a timely way, soon after students complete the VS.

Facilitators need to work through the VS themselves so they can understand and address students’ questions during the debriefing, explore responses to different options, and tie decisions to context and clinical practice. One facilitator (F10, nursing) shared their approach with students, saying, “That's the best in that situation. But let's think if this situation might not have applied.” This facilitator recommended exploring the less-than-ideal responses, probing students to think, “I think learning safely from a less than perfect answer is so powerful that's brought out in facilitation.” The VS may show perfect action, and expert facilitation explores decisions when the situation is not so clear or so simple. This facilitator urged others to ask students, “Where did you disagree with the VS? because that is where learning is richest” (F10 nursing). Learning from mistakes was perceived as an invaluable part of the learning process.

The facilitators also identified supports that they felt had contributed to successful facilitation. One site held regular team meetings to identify what was working and the challenges facilitators were facing to provide support for preparing for VS facilitation.

The students agreed with much of the facilitators’ comments. Students could easily recognize a skilled or non-skilled facilitator, and they emailed the lead facilitator to say “They're just not doing the debrief right. … the students who know what a debrief is supposed to be and what they're supposed to get out of it. They know that that's where the learning is happening” (F5, medical lab). Students also recognize the value of working through both correct and incorrect pathways. One student (FG1, nursing) noted, “It's not about getting the right answer; it's about thinking about why you would choose to take this path.” Another noted, “It does give you a way of reflecting, and to see what you can do better. What it’s going to mean for your client” (FG4, nursing). Students also noted that when the debriefing group was too large (one was part of a class of 35), they did not have an opportunity to share their thoughts.

#### Debriefing with practice partner

Many sites had invited a practice partner to participate in the VS debriefing as clinical experts and strongly recommended continuing this process. Having someone with current clinical experience participating in the debriefs added greatly to the credibility of the VS scenario and the decisions healthcare personnel were seen making in the VS. The practice partners were able to tell students when they had seen a particular scenario in practice and verified that they would respond the same way as in the VS. The facilitators valued the practice partners’ contributions; they felt the partners added to and supported their role. One facilitator (F5, medical lab) noted, “having an industry (practice) partner there makes it more real… I think it's a critical part.”

Another facilitator noted that the practice partner’s presence added weight to the facilitator’s comments, enriched the discussion and helped students see the complexity of dealing with healthcare professionals and patients. This facilitator also noted that when a practice partner was present, they could role model respectfully disagreeing with another’s views. Facilitators also acknowledged that having a practice partner present could be challenging for reasons of time and cost.

The benefits of having the practice partner participate in the VS learning experience were viewed as a two-way street. The facilitators reported that their practice partners found the VS scenarios authentic and the learning process valuable. One facilitator (F2, nursing) commented, “The partners were able to see first-hand what students were learning,” and they had remarked, “Your students are going to have such a better level of understanding and practice prior to coming to our facility.” Another advantage was that participating in the debrief strengthened ties between students, facilitators, and practice partners. They continued to note, “We had an opportunity to discuss and ask questions, and learn a little bit more about their practice, their expectations, as well as the students’ current understanding.”

Facilitators made several recommendations regarding debriefing with practice partners. They emphasized that it is important to prepare the partners for their role in the debriefing, and partners need to be clear on learning objectives and the purpose of the simulation. Partners also need to be familiar with the technology, the VS platform, and the VS itself to prepare for the debrief. In addition to providing partners with a robust debriefing guide, facilitators suggested creating a package with questions facilitators want students to think about during the VS, including any background information. Another suggestion was to create a VS demonstrating the prebrief and debrief.

Students were unanimous in recommending having a practice partner present, commenting that they valued the “objective perspective” that the practice partner brought to the debriefing, their knowledge of current and best practices, and how practice partners shared their experiences and strategies. One student (FG1, nursing) commented, “This is actually what happens sometimes, and you have to apply what you know about best practice to make it work in in the real-life setting, and that was really helpful for me.”

#### Impact on learning

We identified three sub-themes for the second major theme, Impact on learning. These were: Student outcomes, technology and design, and repetitive play.

#### Student outcomes

Facilitators reported that the VS had a significant impact on student outcomes. The VS helped students by reinforcing learning, identifying gaps in student knowledge, and transferring classroom theory into practice. Facilitators felt the VS developed critical thinking and communication skills such as active listening and encouraged students to ask questions, reflect, and develop empathy. Some of this learning developed because the VS created an opportunity for students to encounter activities that were hard to duplicate in the classroom or experience events that do not often arise in practice but are important to prepare for such as a mass casualty situation. One paramedic facilitator (F7, paramedic) noted, “the virtual [simulation] setting allowed us to teach things that are harder to teach in person without being out there on the road and actually experiencing it.” They continued, “And then all of a sudden, you're in it. In VS you could see the scene.”

Another advantage of the VS was that they allowed students to practice safely and repeatedly, something that is more difficult with in-person simulation. The VS lets students practice “it is closer to real life experience with the safety that they can take risks that they might not take in the real environment” (F10, nursing). The opportunity to repeat the VS increased understanding and learning, “it layered and built” (F2, nursing). Several facilitators commented that they would remind students to play the VS repeatedly in the future.

Another important outcome was that the VS helped students prepare for practice; it primed them for what was to come. The VS enabled students to see healthcare providers fulfilling their roles and to get a glimpse of themselves in those roles in the future. Facilitators noted that the VS showed students how providers communicate with clients and demonstrate empathy and enabled students to see the situation from the patient’s perspective. The VS brought patient situations alive, making the situations seem like a real possibility. The VS helped students to know what to expect and to see what action was taken. Facilitators felt the VSs were good preparation for lab and acted as a bridge to practice. The VSs let students see some of the “what ifs” they will face in practice. One paramedic facilitator (F6, paramedic) noted:

VS helps students get beyond learning to pass the test to learning the material to engage in practice. [They gain] the understanding that we build on the foundation to get you road ready. VS encourages them to understand that there is that next level of our education which is now practice.

The VS also contributed to learning by exposing students to measurable “doses” of experience, where they could tackle situations appropriate to their current level and gain experience with that situation before proceeding to the messy world of clinical practice. One facilitator (F2, nursing) encouraged students to ask themselves, “What am I going to do if…? if I observe this in a workplace, how am I going to set myself up for this? What choice would I make?” Another (F5, medical lab) noted:

Some students commented that they went to the bench and saw the chaos of real life. And then did the VS and it was like, oh, that's all the person is thinking about. Then they did the simulation, the VS, and we're like, okay. And then they go back out into the real life, and they're able to focus a little bit more on what's the important thing in real life as well.

Regarding preparation for practice, the students agreed with facilitators. One explained, “my confidence improved after each session, in terms of being able to confidently go into a clinical situation” (FG1, nursing). A paramedic student (FG4, paramedic) noted:

What we normally do is to recall knowledge. Like, you're not really facing it unless you are in a clinical setting. Now we're having a first-hand knowledge before we get into the clinical field. I figure the more practice I have doing all of these things that I might come across on the road, then the more chance I have to remember my protocols and what I'm supposed to be doing.

#### Technology and design

Facilitators felt strongly that VS design matters. The more realistically portrayed clinical situations, for example, those with live action video of patient situations, gave students an emotional connection to the situation that contributed to their learning and made learning “stick.”

“Students like the fact that they're real people that are portrayed. You know, the patients and the team members, rather than avatars, which only adds to the realism” (F8, nursing). An avatar-based VS that used a chat function was described as less meaningful by students. Another facilitator (F10, nursing) noted:

A key strength of these videos is you don't watch them in the same degree of detachment as you would that was something that was strictly informational. The invitation to become part of this that is reinforced by those questions of, ‘What will you do now?’ just heightens the learning and the commitment and the degree of involvement for the student. It's very powerful.

The VS content needed to be tied closely to course content and learning objectives, and the situation had to be realistic. Overly simplistic VS were viewed as “silly and a little bit insulting…” (F8, nursing).

Students agreed that design influenced their learning. Both facilitators and students emphasized that to have that emotional connection that fosters learning, students need to have an easy, “glitch-free” technical experience with the VS. Technical problems were “incredibly frustrating” and interrupted the flow of the experience and impeded learning. Some students reported that they were assigned VS created on different platforms, and it was challenging to learn how to navigate the different systems. They found some VS difficult to log into and to navigate. One participant (FG5, paramedic) commented, “It's just too difficult to navigate through, and I don't feel like I’m learning as much, or reinforcing my learning as much as I could be if I understood it a little bit more.” In one case, no feedback was provided regarding decisions made, which impaired learning. Students wanted certain features added, such as the ability to pause and bookmark the VS if they could not complete it, and they wanted to be able to back up and repeat a section.

#### Repetitive play

Facilitators noted that some students played the VS more than once, and that there were benefits to this. Students were able to delve more deeply into the situation. They could move beyond simply aiming for the right answer and explore incorrect answers and the rationale for those. After hearing that feedback, some facilitators started thinking about encouraging repetitive play in the prebrief, especially since some students did not realize they were allowed to play the VS more than once.

About one-third of the students reported replaying the VS. Some students replayed the VS because they had technical problems, while other students replayed because they wanted to improve their scores or because they found value in playing a second time. One student (FG1, nursing) noted:

I just took an opportunity to think about not what the right answer was, but why I was making the decision, and what was going further down the pipe. What are going to be the ramifications of the decision I was making. And when I had to redo that one a second time. That's when the information really landed. And I thought, oh, okay, now I really get it.

## Discussion

As the demand for highly skilled healthcare workers increases, educators are developing and using VS to effectively prepare students for clinical practice. It is not enough to simply assign VS in a course; VS must be facilitated skillfully. While there are best practices for simulation, facilitating VS has nuances not addressed in the best practice guidelines. With in-person simulation, students are present for the enactment and debrief, while the flexibility and accessibility of VS allows for different ways to enact and debrief such as in-person, groups, individual, virtual, facilitated, and non-facilitated. The purpose of this pan-Canadian study was to gain insight into the impact of VS and in particular the VS facilitator’s role, experiences, and needs from both the facilitator and student perspective. Facilitators and students from across Canada and three healthcare programs participated in the study. The VS were delivered on a range of different platforms. The findings from both facilitators and students in this study were strongly in agreement and support earlier research that highlights the importance of skilled facilitation in VS [5, 26, 27].

Badowski and Wells-Beede [1] raised the question of whether formal training in simulation pedagogy is needed or if it is sufficient to learn “on the job,” questioning how likely facilitators would be to use evidence-based practices without training. Results from this study demonstrate that the role of the facilitator is complex, facilitators must manage events and issues as they arise, using their knowledge and skills to create a safe, stimulating learning environment, a finding supported by de Wijse-van Heeswijk [7]. We learned that the facilitator plays a vital role, and that it is not sufficient to be trained only in in-person simulation, facilitators require training in all stages of the VS cycle. Additionally, it is important for facilitators to understand how the VS, and in particular the debriefing, align with program needs and learning objectives to help students make those connections. To prepare, facilitators need to work through all branches of the VS themselves to understand the students VS experience. Facilitators told us that students learn when correct pathways are reviewed, but that there is also a tremendous amount of learning that arises from exploring the “what ifs” or incorrect decisions and the rationale for those. Perfect action may be demonstrated in the VS; however, facilitation that explores decision-making when the situation is not so clear and simple augments learning.

In connection with exploring, we also learned that there is value in playing the same VS more than once. Students learned from their mistakes, and repetition encouraged reflection and deliberate practice. A recent study by Fogg et al. [9] supports this finding, suggesting that targeted repetitive practice is needed to develop prioritizing and decision-making skills in nursing students. While further research is needed on repetitive play, its impact on cognitive load, and its role in deliberate practice, there are potential advantages for facilitators to encourage students during the prebrief, to play a VS more than once, and to provide a rationale for that action. We also learned that facilitators found debriefing is most effective when done in a timely manner after the VS, supporting earlier findings [19].

Initial training and peer-reviewed facilitator guides are important for novice facilitators, as is ongoing support. Mentoring by experienced facilitators helps to encourage skill development and novice facilitator satisfaction. Another approach is to ask one or more peers to review each other’s debriefing sessions and to provide feedback, a process known as “debriefing the debriefer.” One site provided regular team meetings where facilitators were encouraged to explore what was effective and where they needed help, and this was perceived as very useful. The suggestion to develop a VS on facilitating was made for the team to consider. Our findings support the need to train and support facilitators, a finding also identified in a large, recent scoping review by Park et al. [22].

Another important finding from this study was the value of having a practice partner participating in VS debriefing. Both facilitators and students felt the practice partner added credibility to the learning experience and validated both the VS scenarios and the decision-making observed in the VS. This finding is similar to a finding identified in a recent qualitative study by Holmes and Mellanby, [12] who reported that credibility is associated with having background experience in the clinical topic under study. We found industry/practice partner participation reinforced to the student that what they were learning was relevant, confirmed that the VS experience occurs in real life, and that learning gained from the VS is essential for workplace readiness. The combination of clinical credibility and facilitation skills contributed significantly to student learning in the current study.

Other benefits existed from having a clinician present; it strengthened the connection between students, facilitators, and clinical sites. Practice partners also felt more aware of and involved in current healthcare education. Further, practice partners realized that the VSs could potentially be useful to clinical sites as interactive professional development tools. Wood et al. [31] noted that meaningful work-integrated learning demands a strong partnership between students, educational institutions, and clinical or field partners. Practice partners need to be well-prepared by facilitators for their role and instructed in debriefing techniques. While of definite value, having practice partners present has time, logistical, and cost implications.

Another important finding was that both the facilitators and students observed that learning was influenced by VS design: the emotional connection students made to the clinical situation in the VS was largely the result of realistic scenarios and live action video. This finding is important to facilitators who choose the type of VS used by their students. In this study, the avatar-type VS were described as less effective. Students valued seeing a “real” situation that engaged them, encouraged them to think, and helped prepare them for practice because they could better visualize their future practice. In a study conducted to explore to what extent practicing nurses transferred simulation learning 3 years later to their practice, the participants noted that the “visually memorable nature” of the simulations was a key factor [15]. Peddle et al. [23], in a study of virtual patients, noted that when scenarios were authentic, students felt they were more likely to recall that information at a later date. These findings have important implications for VS design,we need to better understand what design best aligns with different levels of learning objectives.

Students in this study felt that they benefited by playing VS before clinical placements, a finding supported by Turner et al. [27]. Students felt the VS took them, after classroom instruction and lab skill practice, one step closer to clinical practice. That finding was also noted by both Bridge et al. [2] and Diaz et al. [8] who observed that careful, systematic use of VS could potentially reduce clinical hours. Another benefit of the VS was that it provided an opportunity for students to experience complex, less frequently observed clinical situations. Further, the VS allowed students to perform skills before encountering the “noise” of the clinical setting. Students could focus, learn, and practice, and this prepared them for clinical practice, an important finding for educators.

While the results of this study point strongly to the advantages of skilled VS facilitation, further research is needed. Facilitation may be required for VS that cover high-stress, sensitive content, however the level of facilitation required for low-stress scenarios is unknown [30]. It is possible that self-debriefing supported by reflective questions might be sufficient. More research on design and its impact on achieving different levels of learning outcomes is also needed. The opportunity for repetitive play and its role in developing deliberate practice also needs to be further explored.

Lastly, as many earlier studies have suggested, students need a smooth, easy technical experience for optimal learning [17, 29]. In their large systematic review of VS, Foronda et al. [10] reported that technical problems were an issue resulting in student anxiety, frustration, and dissatisfaction with the learning experience. Usability testing for all new VS is essential before embedding a VS in the curriculum. In addition, this finding highlights the need for facilitators to include information about the technology of the VS in the prebrief to mitigate potential challenges students might face. In the Virtu-WIL program, some of the seven platforms were easier to use than others, and the ones that were more difficult to navigate required increased attention in the prebrief to effectively prepare students for the experience.

### Limitations

Our study was conducted with 10 VS facilitators of varying experience from three healthcare programs and 21 students from two programs who played VS on a variety of platforms. While we aimed to include students from all three programs, the majority were from nursing, reflecting the actual population of VS users in the Virtu-WIL project. Because we used purposive and convenience sampling, participants may have volunteered because of their enthusiasm for the project which may have positively influenced their responses to the interview questions. And while interviewers did not work at the students’ institutions, it is possible students may have responded to questions in a way they hoped would please the interviewer. The research team’s experience with VS is another potential limitation, although we took steps at each stage of the research process to minimize bias. As with other qualitative research, the results are not intended to be generalized; however, other groups may be able to transfer the findings to their practice because of the range of platforms used.

## Conclusion

This study examined the role and experience of VS facilitators and students’ learning through completing a minimum of three VS and a corresponding debrief. This research highlights the importance of skilled facilitation in all stages of VS pedagogy. The results of this study suggest that when VS is well facilitated, it helps students achieve learning outcomes, and they feel increased readiness to practice. We also learned that practice partners can play an important role in debriefing sessions, and that this practice strengthens connections between education and the practice site. More research is needed to help us understand the unique role of the facilitator when using VS with students.

## Data Availability

Access to transcripts is available on request.

## Abbreviations

VS: Virtual simulation
CICan: Colleges and Institutes Canada
Virtu-WIL: Work-integrated learning
